# Draft Genome Sequence of Enterobacter mori AYS9, a Potential Plant Growth-Promoting Rhizobacterium

**DOI:** 10.1128/mra.01008-22

**Published:** 2022-11-03

**Authors:** Ayomide Emmanuel Fadiji, Ayansina Segun Ayangbenro, Akinlolu Olalekan Akanmu, Olubukola Oluranti Babalola

**Affiliations:** a Food Security and Safety Focus Area, Faculty of Natural and Agricultural Sciences, North-West University, Mmabatho, South Africa; University of Arizona

## Abstract

Here, we report the draft genome sequence of Enterobacter mori AYS9, a rhizobacterium isolated from the rhizosphere of sorghum plants in South Africa. The genome sequence comprised 4,852,175 bp and exhibited a GC content of 55.5% and 4,567 genes, with 4,453 coding sequences, 3 rRNAs, 64 tRNAs, and 1 CRISPR.

## ANNOUNCEMENT

Members of Enterobacter, a genus of Gram-negative bacteria, have been isolated from different habitats and reported to exhibit close associations with different environments and hosts, such as water, plants, humans, and animals. Many species of this genus are associated with the rhizosphere and endosphere of plants (1–3). Enterobacter mori, one of the most common species associated with plants, has been reported to possess multiple plant growth-promoting characteristics (4). Several studies have reported the plant growth-promoting traits of Enterobacter mori in various plants and crops, such as white mulberry (5), maize (3), and peanut (6).

Soil samples were collected on a teaching and research farm of the North-West University, South Africa (25°47′25.24056″S, 25°37.30′8.17464″E), as described by Sha’arani et al. (7). A pure rhizospheric bacterium was isolated from 10 g soil tightly adhering to the root of a sorghum plant by serial dilution and plating on Luria-Bertani (LB) agar plates as described by Majeed et al. (8). After subculturing, the single colony was confirmed on LB agar through morphological and biochemical tests. The isolate, Enterobacter mori strain AYS9, was observed to possess plant growth-promoting traits, such as phosphate solubilization (9) and the production of siderophore (10), indole-3-acetic acid, and ammonia (11, 12). The discovery of these traits encouraged us to proceed with whole-genome sequencing of this strain.

Genomic DNA was extracted from the pure culture on solid agar using a soil microbe extraction kit (Zymo Research, USA). The genome of strain AYS9 was sequenced at Novogene Co. Ltd., Singapore. A paired-end (PE) sequencing library was prepared from the DNA sample using the Illumina Nextera DNA Flex library preparation kit. The PE Illumina library was loaded onto the NovaSeq 6000 (2 × 150 bp) instrument for cluster generation and sequencing. Whole-genome sequencing was visualized using KBase (13), which yielded 1,979,370,600 reads, representing 394x genome coverage. The read quality was examined using FastQC v43.1.0.4 (14). Trimmomatic v1.2.14 was used to filter out the adapter regions and low-quality reads from the data (15). After the filtering and trimming steps, a total of 1,914,044,780 reads were obtained. The genome assembly was created using SPAdes v1.2.4 (16). A total assembly size of 4,852,175 bp, with 34 contigs, 55.5% GC content, an *N*_50_ value of 750,407 bp, and an *L*_50_ value of 2, was obtained. This file was then used to perform BLAST assessment against GTDB-Tk v1.7.0 (17).

Annotation for the prediction of gene functions was carried out using the NCBI Prokaryotic Genome Annotation Pipeline (PGAP) (18). The result revealed a total of 4,023 genes, 4,453 coding DNA sequences (CDSs), 34 pseudogenes, 80 noncoding sequences, 3 rRNAs, 67 tRNAs, and 7 noncoding RNAs (ncRNAs).

### Data availability.

The whole-genome sequence of Enterobacter mori strain AYS9 has been deposited at DDBJ/ENA/GenBank under the accession number JAOBNB000000000. The version described in this paper is version JAOBNB000000000. The raw reads are available under the BioProject accession number PRJNA877817 and the BioSample accession number SAMN30723749. The sequence data obtained in this work have been deposited in the NCBI Sequence Read Archive under the accession number SRR21481309.
